# 

**Published:** 1882-10

**Authors:** 


SURG GENLS OFFS

Vol. XIX. | October, 1882. | No. 3.

### The Bistoury.

Thad S. Up de Graff, M.D.
EDITOR.

ELMIRA, N.Y.

